# Irisin Levels are Not Affected by Physical Activity in Patients with Anorexia Nervosa

**DOI:** 10.3389/fendo.2013.00202

**Published:** 2014-01-06

**Authors:** Tobias Hofmann, Ulf Elbelt, Anne Ahnis, Peter Kobelt, Matthias Rose, Andreas Stengel

**Affiliations:** ^1^Division for General Internal and Psychosomatic Medicine, Charité Center for Internal Medicine and Dermatology, Charité – Universitätsmedizin Berlin, Berlin, Germany; ^2^Division for Endocrinology, Diabetes and Nutrition, Charité Center for Internal Medicine with Gastroenterology and Nephrology, Charité – Universitätsmedizin Berlin, Berlin, Germany

**Keywords:** brown adipose tissue, energy expenditure, exercise, FNDC5, myokine, SenseWear™ armband

## Abstract

Irisin was recently identified as muscle-derived hormone that increases energy expenditure. Studies in normal weight and obese subjects reported an increased irisin expression following physical activity, although inconsistent results were observed. Increased physical activity in a subgroup of patients with anorexia nervosa (AN) complicates the course of the disease. Since irisin could account for differences in clinical outcomes, we investigated irisin levels in anorexic patients with high and moderate physical activity to evaluate whether irisin differs with increasing physical activity. Hospitalized female anorexic patients (*n* = 39) were included. Plasma irisin measured by enzyme-linked immunosorbent assay and locomotor activity were assessed at the same time. Patients were separated into two groups (*n* = 19/group; median excluded): moderate and high activity (6331 ± 423 vs. 13743 ± 1047 steps/day, *p* < 0.001). The groups did not differ in body mass index (14.2 ± 0.4 vs. 15.0 ± 0.4 kg/m^2^), irisin levels (558.2 ± 26.1 vs. 524.9 ± 25.2 ng/ml), and body weight-adjusted resting energy expenditure (17.6 ± 0.3 vs. 18.0 ± 0.3 kcal/kg/day, *p* > 0.05), whereas body weight-adjusted total energy expenditure (46.0 ± 1.4 vs. 41.1 ± 1.1 kcal/kg/day), metabolic equivalents (METs, 1.9 ± 0.1 vs. 1.7 ± 0.1 METs/day), body weight-adjusted exercise activity thermogenesis (1.8 ± 0.5 vs. 0.6 ± 0.3 kcal/kg/day), duration of exercise (18.6 ± 4.7 vs. 6.2 ± 3.1 min/day), and body weight-adjusted non-exercise activity thermogenesis (21.6 ± 1.0 vs. 18.8 ± 0.8 kcal/kg/day) were higher in the high activity compared to the moderate activity group (*p* < 0.05). No correlations were observed between irisin and activity parameters in the whole sample (*p* > 0.05). In conclusion, the current data do not support the concept of irisin being induced by exercise, at least not under conditions of severely reduced body weight like AN.

## Introduction

Irisin is a recently identified muscle-derived hormone produced by cleavage from fibronectin type III domain containing 5 (FNDC5) (1). The expression of FNDC5 is induced by the peroxisome proliferator-activated receptor γ (PPARγ) coactivator 1α (PGC-1α) that has been demonstrated to play an important role in the expression of uncoupling protein 1 (UCP1), a key factor of thermogenesis in brown adipose tissue (2). Irisin drives the browning of subcutaneous white adipose tissue by stimulating the expression of UCP1 in beige fat cells (1). These beige fat cells have been shown to be a cell type distinct from white or brown fat cells being located in white adipose tissue (3) and exhibiting properties of brown adipose tissue which – for long thought to exist only in newborns – was recently also detected in adult humans (4, 5) and demonstrated to be activated in response to cold exposure (4). Besides its occurrence in skeletal muscle, irisin was detected – although in much smaller quantities – in several human tissues containing smooth muscle as well as heart muscle, and organs including liver, kidney, and lung (6). Additionally, this peptide is also present in adipose tissue (7, 8) and rodent cerebellar Purkinje cells (9). Lastly, irisin was detected in the circulation (1, 6, 10, 11) with decreased levels in patients with type 2 diabetes mellitus (10).

In their initial study, Boström et al. described an increase of circulating irisin levels following physical activity in mice and in male subjects after a 10-week endurance exercise program (1). In a subsequent study, elevated circulating irisin levels were observed following acute exercise consisting of 80-m sprints, while this effect was weakened after a 8-week sprint training program completed by young healthy men (6). However, one study detected a modest 30% higher FNDC5 mRNA expression only in a subgroup of older male subjects after an endurance training program compared to sedentary controls, whereas no changes were observed in a large sample of younger adults (12). While data on an exercise-dependent production of irisin in muscle and its secretion into the circulation are inconsistent in humans, an association of plasma irisin levels with body mass index (BMI) seems to be a robust finding. Extending the results of a previous study that showed a trend toward a correlation of circulating irisin with BMI in subjects with a BMI range from 20 to 48 kg/m^2^ (6), we previously reported a positive correlation of plasma irisin levels with BMI over a very broad spectrum of body weights with BMIs ranging from 8 to 85 kg/m^2^ as well as positive associations with fat mass (FM), fat-free mass (FFM), and body cell mass (BCM) (11). One previous study on the association of irisin and energy expenditure reported that irisin levels correlated with 24-h energy expenditure measured by indirect calorimetry in overweight and obese post-menopausal women only in a subgroup of subjects whose energy expenditure was higher than predicted by an FFM-based equation (13), whereas it did not hold true when the whole sample was analyzed.

In light of these data, more research is needed in order to characterize the regulation of irisin under conditions of different activity patterns and to establish its possible role as a novel muscle-derived and exercise-induced hormone. In contrast to its potentially beneficial effect as health-promoting hormone in overweight or obese patients, the role as an exercise-induced and energy-dissipating myokine might be an additional biological mechanism explaining worse clinical prognosis and higher relapse rates of hyperactive anorexic subjects. Furthermore, anorexia nervosa (AN) might be viewed as a mirror image of obesity and thus investigation of this disease likely contributes to the understanding of weight disorders in general. Therefore, in the present cross-sectional study we investigated the relationship of circulating irisin levels with measures of physical activity and different aspects of energy expenditure in anorexic patients, since subjects suffering from AN are known to display very different activity patterns encompassing a relevant subgroup of hyperactive in contrast to moderately active patients (14–16).

## Subjects and Methods

### Subjects

We recruited 39 anorexic female inpatients at admission to their treatment in the Division for General Internal and Psychosomatic Medicine at Charité – Universitätsmedizin Berlin. Inclusion criteria were a BMI of <18 kg/m^2^ and fulfillment of ICD-10 (International Statistical Classification of Diseases and Related Health Problems of the World Health Organization, 10th revision) criteria (17) for typical or atypical AN. Atypical AN is defined as a disorder meeting some criteria of the typical form (BMI below 17.5 kg/m^2^, self-induced weight loss, body image distortion, and endocrine disorder most often reflected by secondary amenorrhea) without fulfilling all key symptoms (17). The restricting subtype of AN according to the fourth edition of the Diagnostic and Statistical Manual of Mental Disorders (DSM-IV) differs from the purging subtype by the absence of binge-eating or purging behavior such as self-induced vomiting or misuse of appetite suppressants, thyroid hormones or laxatives, diuretics, and enemas (18).

According to their locomotor activity, anorexic patients were separated into two groups by dividing the patients at the median step count resulting in a moderate activity and a high activity group (*n* = 19/group, one patient with the median value was excluded). Additionally, since the restricting subtype was shown to be associated with excessive exercise (19, 20), calculations were also performed for three subgroups of AN: typical restricting, typical purging, and atypical AN.

Patients with current pregnancy or psychotic disorders and an age of <18 years were excluded. The investigations were conducted according to the Declaration of Helsinki. All patients gave written informed consent and the study was approved by the institutional ethics committee of the Charité – Universitätsmedizin Berlin (protocol number: EA1/114/10).

### Laboratory analysis of irisin plasma levels

All blood samples were collected from a forearm vein between 7 and 8 a.m. after an overnight fast during the period of the data collection on physical activity. Participants were allowed to drink small amounts of water but were advised not to smoke or exercise before blood withdrawal. The blood was collected in pre-cooled EDTA tubes prepared with aprotinin (1.2 Trypsin Inhibitory Unit per 1 ml blood; ICN Pharmaceuticals, Costa Mesa, CA, USA) for peptidase inhibition. The tubes were placed back on ice immediately after blood withdrawal and then centrifuged at 4°C for 10 min at 3000 × *g*. Plasma was separated and stored at −80°C until further processing. At the day of the measurement, samples were diluted 1:10 and irisin plasma levels were analyzed using a commercial enzyme-linked immunosorbent assay (ELISA, catalog # EK-067-16, Phoenix Pharmaceutical, Inc., Burlingame, CA, USA). All samples were processed in one batch (intra-assay variability <5%).

### Measurements and calculation of activity and energy expenditure

Weight and height were assessed on the day of the blood collection. Weight was measured to the nearest 0.1 kg and height to the nearest 0.5 cm. The BMI was calculated as kilogram per square meter. Physical activity data were continuously measured for 3 days (Friday to Sunday) during inpatient treatment using a portable armband device (SWA; SenseWear™ PRO3 armband; BodyMedia, Inc., Pittsburgh, PA, USA). One day was included in the data analysis if the minimum duration of data acquisition was 20 h and 30 min/day. Patients were not activity restricted during the measurement. Activity displayed by individuals is suggested to be affected only in part deliberately with a high predictive value of the activity level under sedentary conditions for daily life activity (21). Although activity patterns under conditions of hospitalization might differ from those in daily life, activity habits are likely to persist during inpatient treatment if activity is not restricted due to a genetic influence on physical activity (22).

The SWA uses a multisensory array including sensors measuring heat flux, galvanic skin response, skin temperature, near-body ambient temperature, and a two-axis accelerometer. Step counts were measured by the accelerometer and directly taken for analyses. Obtained data were analyzed using a generalized proprietary algorithm developed by the manufacturer (SenseWear™ Software, Version 6.1, BodyMedia, Inc.) and prepared for statistical analysis.

Total energy expenditure (TEE) consists of resting energy expenditure (REE), thermic effect of food (TEF), and activity thermogenesis. Activity thermogenesis can be further separated into the two components exercise-related activity thermogenesis (EAT, deliberate physical exercise, sports) and non-exercise activity thermogenesis (NEAT, spontaneous daily physical activity) (23). Energy expenditure of more than five metabolic equivalents (METs) of a task (a physiological measure expressing the energy cost of physical activities) was classified as EAT, whereas energy expenditure of up to five METs was classified as NEAT adapting the findings of Ainsworth et al. (24) and as described before by our group (25). While TEE, EAT, and duration of exercise were directly received from the proprietary algorithms of the SWA, NEAT was calculated according to the equation: NEAT = TEE − TEF − REE − EAT. TEF was estimated as 10% of TEE and calculated as TEE × 0.1 (26). REE, required for calculation of NEAT, was calculated using weight-/group-specific REE prediction equations provided by Müller et al. (27) since REE cannot be directly determined by the SWA.

### Statistical analysis

Distribution of the data was determined by using the Kolmogorov–Smirnov test. Data are expressed as mean ± SEM. Differences between groups were calculated using the *t*-test. For the comparison of the subtypes of AN differences between groups were calculated using one-way analysis of variance (ANOVA) followed by Tukey *post hoc* test. Correlations were determined by Pearson’s or Spearman’s analysis depending on the distribution of the data. Differences between groups were considered significant when *p* < 0.05. All statistical analyses were conducted using SigmaStat 3.1.

## Results

### Irisin plasma levels do not depend on activity and energy expenditure

**Figure 1:**
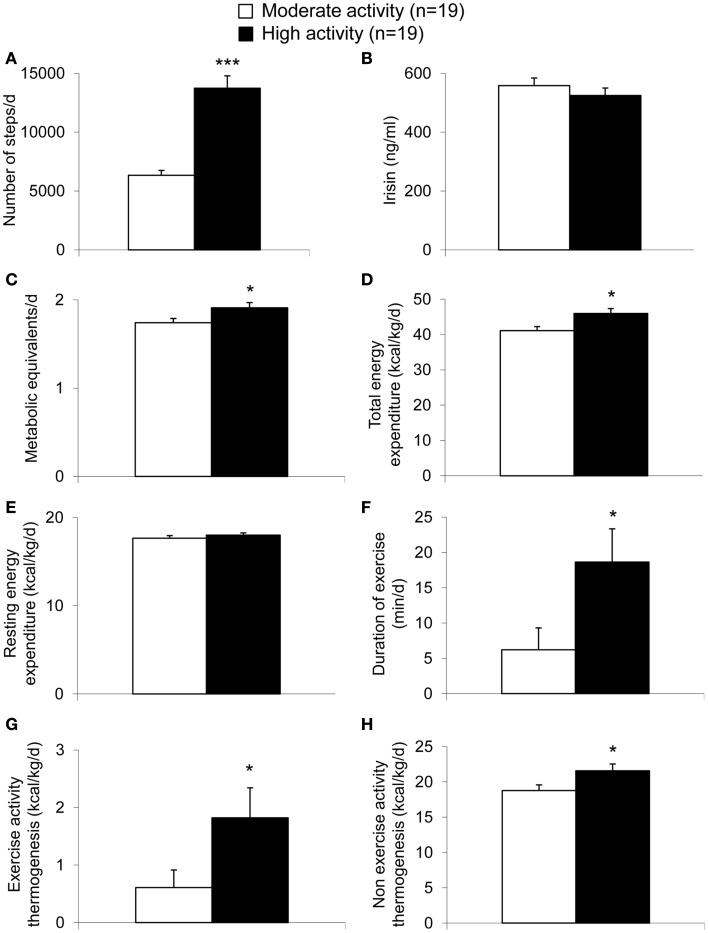
**Parameters of energy expenditure in anorexic patients with moderate and high physical activity**. Per definition, the high activity group showed a higher number of steps compared to the moderate activity group **(A)**. Irisin levels did not differ between the two groups **(B)**. The metabolic equivalents per day **(C)** as well as body weight-adjusted total energy expenditure **(D)** were higher in the high compared to the moderate activity group, while no differences were observed for body weight-adjusted resting energy expenditure **(E)**. Duration of exercise **(F)**, body weight-adjusted exercise activity thermogenesis **(G)**, and body weight-adjusted non-exercise activity thermogenesis **(H)** were higher in the high compared to the moderate activity group. Data are expressed as mean ± SEM. *p < 0.05, **p < 0.01, and ***p < 0.001 in the high vs. moderate activity group (*n* = 19/group).

Per definition, the moderate activity and the high activity group differed significantly in the number of steps performed per day (*p* < 0.001; Figure 1A). However, irisin plasma levels were not different between these two groups (*p* = 0.37; Figure 1B). Associated with the higher activity, significant differences were observed for METs per day (*p* = 0.03; Figure 1C) and body weight-adjusted TEE (*p* = 0.009; Figure 1D) with higher values in the high compared to the moderate activity group. No differences were detected for body weight-adjusted REE (*p* = 0.38; Figure 1E), whereas duration of exercise (*p* = 0.03; Figure 1F) or body weight-adjusted EAT (*p* = 0.03; Figure 1G) and body weight-adjusted NEAT (*p* = 0.03; Figure 1H) were higher in the high activity compared to the moderate activity group.

**Figure 2:**
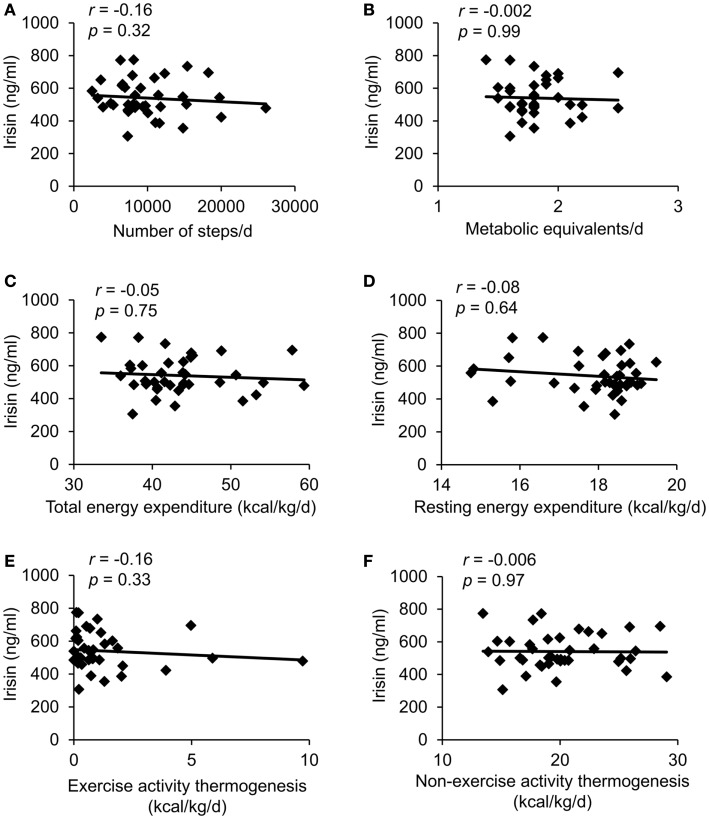
**Correlations between circulating irisin concentrations and number of steps per day (A), metabolic equivalents per day (B), body weight-adjusted total energy expenditure (C), body weight-adjusted resting energy expenditure (D), body weight-adjusted exercise activity thermogenesis (E), and body weight-adjusted non-exercise activity thermogenesis (F)**. Values for *r* and *p* are indicated in each graph, *n* = 39 patients.

In the whole sample no correlation was observed between plasma irisin levels and the number of steps as a measure of activity (Figure 2A), the amount of METs (Figure 2B) and other parameters of energy expenditure, namely body weight-adjusted TEE (Figure 2C), body weight-adjusted REE (Figure 2D), body weight-adjusted EAT (Figure 2E), and body weight-adjusted NEAT (Figure 2F).

### Irisin plasma levels show a negative correlation with components of energy expenditure in patients with restricting type anorexia nervosa only

After division of the sample into subgroups according to the type of AN, namely purging type (*n* = 10), restricting type (*n* = 20), and atypical (*n* = 9) AN, the three groups did not differ in terms of age and BMI (*p* > 0.05; Table 1). As shown in Table 1, no differences among these three groups were observed for irisin, number of steps per day, METs per day, and the different components of energy expenditure (*p* > 0.05).

**Table 1:** **Parameters of energy expenditure according to the subtype of anorexia nervosa**.

Parameter	Group		
	Purging type AN (*n* = 10)	Restricting type AN (*n* = 20)	Atypical AN (*n* = 9)
Age (years)	28.6 ± 3.1	27.8 ± 2.4	25.4 ± 1.7
Body mass index (kg/m^2^)	14.1 ± 0.7	14.3 ± 0.4	15.7 ± 0.6
Irisin (ng/ml)	578.5 ± 41.5	522.8 ± 21.8	536.9 ± 38.6
Number of steps per day	11011 ± 2195	9804 ± 939	9307 ± 1567
METs per day	1.85 ± 0.12	1.83 ± 0.04	1.80 ± 0.08
TEE (kcal/kg/day)	43.7 ± 2.7	43.4 ± 0.9	43.9 ± 2.1
REE (kcal/kg/day)	17.7 ± 0.4	17.9 ± 0.3	18.0 ± 0.2
Duration of exercise (min/day)	17.5 ± 8.9	9.7 ± 2.4	12.4 ± 6.2
EAT (kcal/kg/day)	1.8 ± 1.0	0.9 ± 0.2	1.2 ± 0.6
NEAT (kcal/kg/day)	19.8 ± 1.5	20.3 ± 0.8	20.2 ± 1.7

Data are expressed as mean ± SEM, *p* > 0.05. AN, anorexia nervosa; EAT, exercise activity thermogenesis; MET, metabolic equivalent; NEAT, non-exercise activity thermogenesis; REE, resting energy expenditure; TEE, total energy expenditure.

When investigating the three subgroups separately, irisin levels showed a negative correlation with the number of steps per day, duration of exercise, and body weight-adjusted EAT in patients with restricting type AN (*p* < 0.05; Table 2), whereas no correlations of plasma irisin and components of energy expenditure were observed for the subgroups of purging type and atypical AN (*p* > 0.05; Table 2).

**Table 2:** **Correlation of irisin with parameters of energy expenditure according to the subtype of anorexia nervosa**.

Parameter	Group		
	Purging type AN (*n* = 10)	Restricting type AN (*n* = 20)	Atypical AN (*n* = 9)
Number of steps per day	*r* = −0.055; *p* = 0.865	*r* = −0.465; *p* = 0.039*	*r* = 0.283; *p* = 0.434
METs per day	*r* = −0.043; *p* = 0.892	*r* = −0.171; *p* = 0.464	*r* = 0.332; *p* = 0.356
TEE (kcal/kg/day)	*r* = −0.139; *p* = 0.681	*r* = −0.251; *p* = 0.280	*r* = 0.333; *p* = 0.356
REE (kcal/kg/day)	*r* = −0.091; *p* = 0.785	*r* = 0.000; *p* = 0.997	*r* = −0.317; *p* = 0.381
Duration of exercise (min/day)	*r* = −0.006; *p* = 0.973	*r* = −0.524; *p* = 0.018*	*r* = 0.151; *p* = 0.676
EAT (kcal/kg/day)	*r* = −0.018; *p* = 0.946	*r* = −0.519; *p* = 0.019*	*r* = 0.150; *p* = 0.676
NEAT (kcal/kg/day)	*r* = −0.127; *p* = 0.707	*r* = −0.147; *p* = 0.529	*r* = 0.367; *p* = 0.308

AN, anorexia nervosa; EAT, exercise activity thermogenesis; MET, metabolic equivalent; NEAT, non-exercise activity thermogenesis; REE, resting energy expenditure; TEE, total energy expenditure. Values for *r* and *p* are indicated in each row, *p < 0.05.

## Discussion

Irisin is a newly defined myokine that increases energy expenditure by stimulating the expression of UCP1 and thus the browning of white adipose tissue (1). In addition, it has been shown that irisin plasma levels are increased in response to different types of exercise (1, 28, 29). Consequently, irisin was proposed to mediate some of the health-promoting effects of physical activity. In turn, irisin could be an additional biological mechanism contributing to the course of the disease and therefore the prognosis in highly active patients suffering from AN (30, 31). However, in rejection of our initial hypothesis, we did not detect differences in irisin plasma levels between anorexic patients with high vs. moderate locomotor activity as determined by average daily step count. When investigating the whole cohort, we also did not observe any correlations of circulating irisin with physical activity, TEE or other parameters of energy expenditure, in particular EAT. Several points could contribute to this lack of correlation of irisin with measures of locomotor activity and energy expenditure in our study.

First, Timmons et al. reported that they failed to detect a strong increase of the irisin precursor, FNDC5 after exercise in a population of younger adults, whereas only in a subgroup of older subjects an increase of FNDC5 mRNA expression after exercise was observed (12). Since our data were collected in a population of young patients with a mean age of 27, the results could underline the hypothesis that exercise-related effects on irisin expression are associated with age and only (or predominantly) observed in elderly subjects.

Second, irisin is a cleavage product of FNDC5 expressed mainly in skeletal muscle. Irisin plasma levels have been shown to be associated with biceps circumference as a marker of muscle mass (6, 11). In addition, using bioelectrical impedance analysis (BIA) in a previous study, we showed a positive correlation of irisin with FFM and BCM (the measure best reflecting muscle mass using BIA) (11). Since patients suffering from AN have greatly diminished muscle mass as a consequence of malnutrition and weight loss, the total muscle mass might be too low in our patient population (BCM 15.54 ± 0.43 kg; FFM 37.02 ± 0.66 kg) to detect differences in the levels of the circulating myokine, irisin.

Third, in line with our finding of a lacking association between energy expenditure and circulating irisin, one recent study did not detect a correlation of plasma irisin levels with 24-h energy expenditure in post-menopausal women when assessed in a metabolic chamber (13). Subjects in this and our study are in a state of down-regulated sexual hormones that could contribute to the results. Another explanation could be that only exercise with respect to a defined acute or repetitive physical training program exerts significant effects on irisin muscle expression or plasma levels, whereas chronic changes in exercise do not. This might be due to counter-regulatory adaptive changes that have to be further investigated. In addition, since irisin has been shown to initiate the browning of white adipose tissue and thereby eliciting an additional energy-dissipating effect (1, 3), the existence of a reasonable amount of beige adipocytes would be crucial to mediate these effects. However, little is known about the existence of brown and beige adipose tissue in fat-deprived anorexic subjects. The activity of brown fat has been reported to be inversely correlated with BMI with reduced activity in overweight or obese compared to lean young male subjects (4). However, a more recent study did not detect brown fat activity in anorexic patients, whereas it was detectable in constitutionally lean (average BMI 16.2 kg/m^2^) subjects (32). The absence of brown (or beige) fat activity could therefore be another factor contributing to the lacking correlation of irisin with components of energy expenditure.

Fourth, parameters of physical activity were measured using the SenseWear™ armband device shown to be the best estimate of energy expenditure when comparing several monitors of physical activity in healthy volunteers under regular daily life conditions (33). When compared with predictive equations (34), the SenseWear™ armband device and the doubly labeled water method have shown sufficient concordance for the assessment of resting and TEE in healthy volunteers and in overweight subjects with type 2 diabetes mellitus (35, 36). However, this comparison is still pending for subjects with reduced body weight. Therefore, the SenseWear™ armband device might have limitations for the use in anorexic patients. In addition, the measurement method of irisin could contribute to the lacking correlation of physical activity and circulating irisin. Erickson recently commented on the missing validity of the antibodies used to detect irisin in the existing studies since a quantitative western blot analysis has not been conducted yet and thus questioned the results on circulating irisin published so far (37). However, the antibody by Phoenix used in the present study seems to be the most suitable to date since it detects an amino acid sequence that is part of the cleaved irisin protein (full cross-reactivity with amino acids 42–112 of the 112-amino acid polypeptide irisin, while no cross-reactivity was observed with the C-terminal parts of the irisin precursor FNDC5 amino acids 149–196 and 162–209, manufacturer’s information). This contrasts with other antibodies that were raised against the transmembrane sequence of FNDC5 (1, 8) unlikely to occur in the circulation.

Lastly, a recent study suggested that the human FNDC5 gene differs from other species by a mutation in the start codon sequence (38) which may result in a lower translation efficiency (39) and consequently could explain difficulties to translate animal data to humans.

After investigating the whole cohort, we separated the study population according to the type of anorexia (purging type, restricting type, and atypical) since there is evidence for differences in excessive exercise in these subtypes of AN (19, 20, 40, 41). However, irisin levels and all other parameters of energy expenditure assessed did not differ between these groups. Similarly to the whole sample, in the subgroups purging and atypical AN, no correlation was observed between irisin levels and any parameter of energy expenditure. However, when investigating the patients with restricting type AN separately, irisin levels negatively correlated with the number of steps per day and body weight-adjusted EAT which may indicate that this subgroup is a more homogeneous population of patients. This negative association is unexpected and in contrast to the reported positive association of exercise and circulating irisin for normal and overweight subjects (1, 6, 28). The present finding may point toward a differential regulation of irisin in patients with restricting type AN (*n* = 20) but should be confirmed in a larger cohort of patients.

In summary, irisin plasma levels do not differ in anorexic patients with moderate activity from those with high locomotor activity. Moreover, no association was observed for plasma irisin levels with various parameters of energy expenditure assessed under unrestricted conditions of physical activity in hospitalized patients. Therefore, the current data do not support the concept of irisin being induced by exercise, at least not under conditions of severely reduced body weight like AN. Thus, it is also unlikely that irisin contributes to exceeding energy expenditure in highly active anorexic patients. Further studies are needed to clarify whether the lacking correlation of irisin and physical activity described here is due to a missing association of irisin and exercise under conditions of reduced body weight *per se* or because of a unique characteristic of anorexic patients.

## Author Contributions

Tobias Hofmann, Ulf Elbelt, and Andreas Stengel designed the study; Tobias Hofmann and Andreas Stengel coordinated and supervised the data collection, carried out the statistical analyses and drafted the manuscript. Ulf Elbelt, Anne Ahnis, Peter Kobelt, and Matthias Rose discussed the data and reviewed the article. All authors discussed the results, reviewed, and finalized the manuscript.

## Conflict of Interest Statement

The authors declare that the research was conducted in the absence of any commercial or financial relationships that could be construed as a potential conflict of interest.
